# Mapping the literature of dental hygiene: an update

**DOI:** 10.5195/jmla.2019.562

**Published:** 2019-07-01

**Authors:** Carol L. Watwood, Terry Dean

**Affiliations:** Health Sciences Librarian/Associate Professor, Department of Library Public Services, Libraries, Western Kentucky University, Bowling Green, KY, carol.watwood@wku.edu; Associate Professor and Allied Health Department Head, Western Kentucky University, Bowling Green, KY, terry.dean@wku.edu

## Abstract

**Objective:**

This study updates Haaland’s 1999 dental hygiene mapping study. By identifying core journals and estimating database coverage, it characterizes changes in dental hygiene research and aids librarians in collection development and user education.

**Method:**

Cited references from a three-year (2015–2017) sample of core dental hygiene journals were collected, categorized into five formats, and analyzed by format and publication year according to Bradford’s Law of Scattering. CINAHL Complete, MEDLINE, and EMBASE were surveyed to determine the indexing coverage of cited journals.

**Results:**

The number of cited journal titles increased from 389 in 1999 to 1,675 in 2018. Core Zone 1 titles increased from 5 to 11. Journal article citations increased from 69.5% of all citations in 1999 to 78.4% in the present study, whereas book citations decreased from 18.1% to 5.1%. A newly added category, “Internet sources,” accounted for 8.4% of citations. Overall, 68.6% of citations were 10 years or younger versus 71.4% in 1999. Most Zone 1 and Zone 2 journals were specific to dentistry or dental hygiene.

**Conclusion:**

Notable changes since 1999 were an increased volume of literature and a shift from print to online sources, reflecting improved accessibility of the literature and greater Internet use. From 1999 to 2018, citations to journal articles increased, books decreased, websites appeared, and government publications increased slightly. These findings indicate that dental hygiene research is growing and that indexing coverage for this field has improved dramatically in the past two decades.

## INTRODUCTION

As part of the Medical Library Association (MLA) Nursing and Allied Health Resources Section (NAHRS) project to map the literature of allied health [1], Haaland published a dental hygiene mapping study in 1999 [2] to identify core journals in dental hygiene and determine indexing coverage for these journal titles. In light of changes in the dental hygiene profession and the amount of time passed since the original mapping study, it is prudent to update and reassess the dental hygiene literature to provide useful information for both librarians and dental hygiene professionals.

As noted in Haaland’s article [2], dental hygienists work with dentists to provide services that “include dental prophylaxis, radiography, application of medications, and provision of dental education at chairside and in the community” [3]. While this definition still describes a large portion of the profession, it does not encompass all of the roles currently played by dental hygienists, who also work in academia, industry, public health, and research [4]. In addition, when the original mapping study was published, only 212 accredited US dental hygiene programs were in place, whereas there are now 330 programs with 21 graduate programs. Most programs grant an associate degree, others lead to a bachelor’s degree, and some offer master’s degrees in dental hygiene or dental therapy [5, 6].

In 2013, dental hygiene celebrated 100 years as a profession. Research articles in dental hygiene began appearing in the 1940s, and in the 1960s, dental hygienists began to engage in dental research projects, usually as associates or administrators rather than principal investigators. The first conference on dental hygiene research was held in 1982 [7]. The National Center for Dental Hygiene Research (established in 1993 and now known as the National Center for Dental Hygiene Research & Practice) updated its research agenda twice: in 2007 and 2016 [8]. Of note, in its 2016 research agenda, the American Dental Hygiene Association stated, “[t]he goal of increasing dental hygienists’ participation in research is to grow beyond reliance on research originating from other disciplines and, instead, build upon existing research so the knowledge base can emerge from within dental hygiene itself” [9]. Another driver of this increased research emphasis was the 2016 Revised Standards for Clinical Dental Practice, which stated that dental hygienists should “[a]ccess and utilize current, valid, and reliable evidence in clinical decision-making through analyzing and interpreting the literature and other resources” [10].

A current mapping study would support such research endeavors and shed light on changes that have shaped applicable literature during the last two decades. Thus, the purpose of this study is, like the original study [2], to identify core journals in the field of dental hygiene and determine the extent of indexing coverage for these journal titles. Its findings could reveal whether the field of dental hygiene is expanding due to increased intellectual contributions and can aid librarians in deciding which journals to collect and which databases to teach and aid dental hygienists in deciding where to submit their manuscripts for publication.

## METHODS

This study used the mapping protocol outlined by NAHRS, as updated in December 2017 [1]. Using EndNote Basic, the author entered cited references from a three-year sample of core dental hygiene source journals from 2015–2017 into Excel 2016 and then sorted and analyzed the references.

Two source journals from the 1999 mapping study [2], the *Journal of Dental Hygiene* and *Probe* (now called *Canadian Journal of Dental Hygiene*), were reused. The third source journal used in Haaland’s study, *Journal of Practical Hygiene*, is no longer published. After consulting with and interviewing the director and faculty of a dental hygiene program, *Dimensions of Dental Hygiene* was selected as a replacement, as it is a widely read journal covering practical issues. All three journals are North American–based titles.

References from each journal article that were published from 2015–2017 in the three source journals were collected. Letters to the editor, editorials, supplementary issues, and brief news items were omitted. Each reference was given a unique identifier and categorized as one of five formats: journal (print or online), book (print or online), government publication (US or foreign, print or online), Internet source (not otherwise categorized as journal, book, or government publication), or miscellaneous (e.g., software, videos, dissertations, conference abstracts). The year of publication of each reference was noted. For journal citations, the cited journal name was recorded as the current or most recent title. The National Library of Medicine (NLM) catalog was used to track title changes.

Spreadsheets of references from each journal were sorted by format and publication year. References from journal titles were listed in order from the most frequently cited to the least frequently cited. The list of journal titles was separated into three zones, each containing about a third of the total cited journal references. According to the NAHRS mapping protocol, these zones correspond roughly to the highly productive, moderately productive, and minimally productive journals in the field [1]. Data were then analyzed using Bradford’s Law of Scattering, which predicts that, in a given discipline, a small number of journals publishes a substantial portion of the literature, with the rest being widely scattered across titles. The number of Zone 1 journals multiplied by *n* equals the number of Zone 2 journals, and the number of Zone 3 journals is predicted to be *n*^2^ × the total number of journals in Zone 1 [10].

The indexing coverage of the cited journals in CINAHL Complete, MEDLINE, and EMBASE was recorded. Coverage was determined from 2018 online title lists that database producers published and recorded as “yes,” “no,” or “selective.”

## RESULTS

A total of 10,901 cited references were recorded: 78.4% were journal articles, 8.4% were Internet sources, 6.5% were government documents, 5.1% were books, and 1.6% were miscellaneous (Table 1). Notably, over half of the references (n=6,299) came from *Dimensions of Dental Hygiene*. Over half (64.8%) of the cited journal articles and most of the cited Internet sources (90.1%) and government publications (85.6%) were published in 2007–2017 (Table 2).

**Table 1:** Format types by source journal and citation frequency

Cited format type	Number of citations in source journals						Totals	

	*JDH*		CJDH*		*DDH*			
			
	No.	%	No.	%	No.	%	No.	%
Journal articles	2,763	76.0%	748	77.3%	5,038	80.0%	8,549	78.4%
Books	193	5.3%	36	3.7%	332	5.3%	561	5.1%
Government documents	280	7.7%	42	4.3%	384	6.1%	706	6.5%
Internet resources	338	9.3%	123	12.7%	452	7.2%	913	8.4%
Miscellaneous	60	1.7%	19	2.0%	93	1.5%	172	1.6%
Total	3,634	100.0%	968	100.0%	6,299	100.0%	10,901	100.0%

*JDH=Journal of Dental Hygiene*.

*CJDH=Canadian Journal of Dental Hygiene* (*formerly, *Probe*).

*DDH=Dimensions of Dental Hygiene*.

**Table 2:** Cited format types by publication year periods

	Cited format type											

Publication year (range)	Journal articles		Books		Government publications		Internet sources		Miscellaneous		Total	

	No.	%	No.	%	No.	%	No.	%	No.	%	No.	%
2012–2017*	3,029	35.4%	247	44.1%	505	71.8%	665	72.1%	104	62.3%	4,550	41.7%
2007–2011	2,512	29.4%	126	22.5%	97	13.8%	166	18.0%	36	21.6%	2,937	26.9%
2002–2006	1,403	16.4%	58	10.4%	43	6.1%	44	4.8%	17	10.2%	1,565	14.4%
1997–2001	665	7.8%	43	7.7%	40	5.7%	30	3.3%	7	4.2%	785	7.2%
1992–1996	328	3.8%	30	5.4%	9	1.3%	5	0.5%	3	1.8%	375	3.4%
Before 1992	611	7.1%	56	10.0%	5	0.7%	6	0.7%	0	—	677	6.2%
Unknown	1	—	0	<0.1%	4	0.6%	6	0.7%	0	—	11	0.1%
Total	8,549	99.9%	560	100.1%	703	100.0%	922	99.9%	167		10,901	99.9%

* Includes in-press materials.

May not add to 100.0% due to rounding.

Zone 1 contained 11 journals that accounted for 33.5% of the cited references (Table 3). All Zone 1 journals were well-established titles, including 7 dental journals, 3 dental hygiene journals, and the *Cochrane Database of Systematic Reviews*. Zone 2 contained 108 journals and included a mixture of dental or oral health journals (61%) and non-dental journals (39%) such as *JAMA*, the *New England Journal of Medicine*, the *American Journal of Public Health, MMWR*, *Academic Medicine*, and the *Journal of Allied Health*. Compared with the 1999 mapping study [2], these results reflect a 13% increase in the citation of non-dental journals.

**Table 3:** Distribution by zone of cited journals and references

Zone	Cited journals		Cited journal references		

	No.	%	No.	%	Cumulative total
1	11	0.7%	2,867	33.5%	2,867
2	108	6.4%	2,851	33.3%	5,718
3	1,556	92.9%	2,831	33.1%	8,549
Total	1,675	100.0%	8,549	100.0%	

According to Bradford’s Law of Scattering, the *n* for this study was 9.8 (108 journals in Zone 2/11 journals in Zone 1), meaning that the number of Zone 3 journals should be 1,056 (9.8^2^ × 11). However, there were 1,556 journals in Zone 3, which was larger than predicted and might be attributed to the growing utilization of online-only journals.

CINAHL Complete, MEDLINE, and EMBASE indexed most of the resources in all three zones, but no single database indexed all of the resources (Table 4). EMBASE had slightly better coverage of Zone 1 and Zone 2 resources than MEDLINE, followed by CINAHL Complete.

**Table 4:**
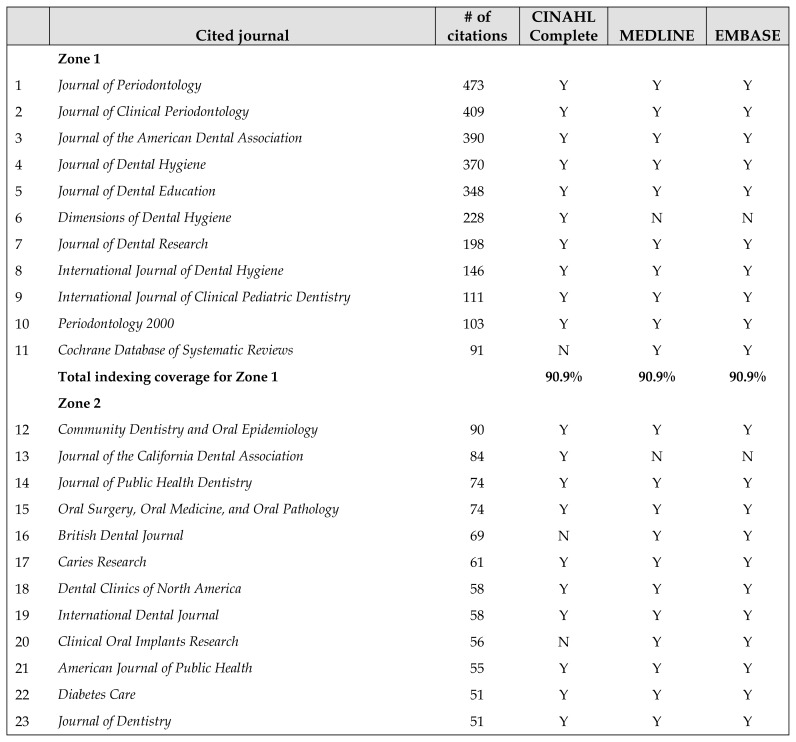

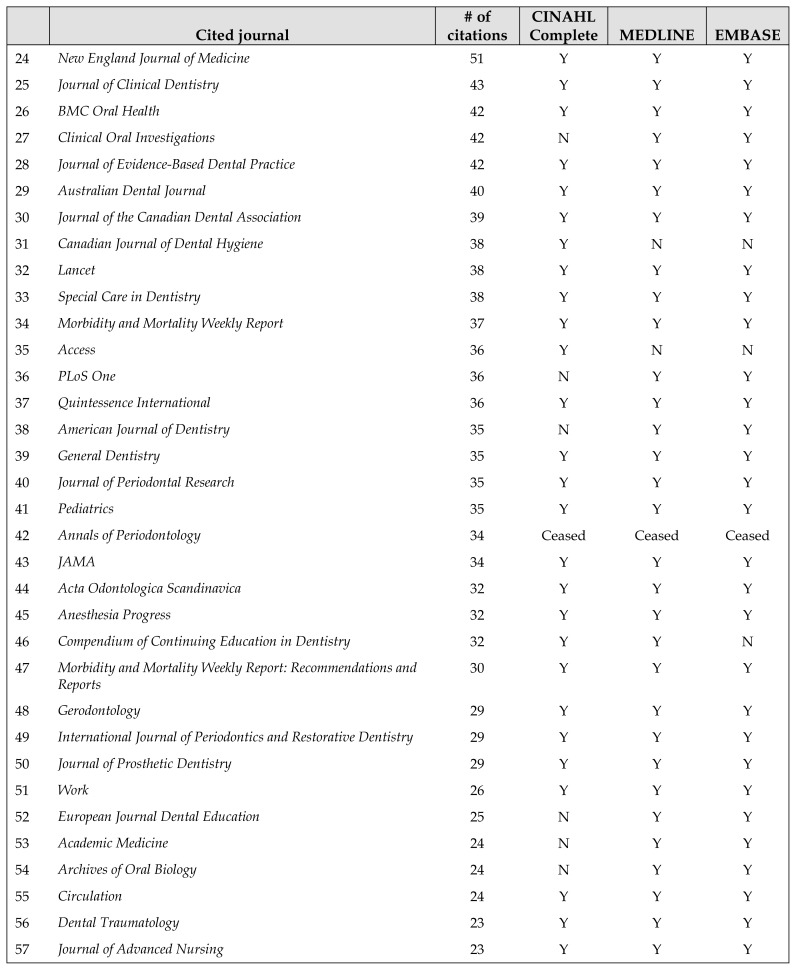

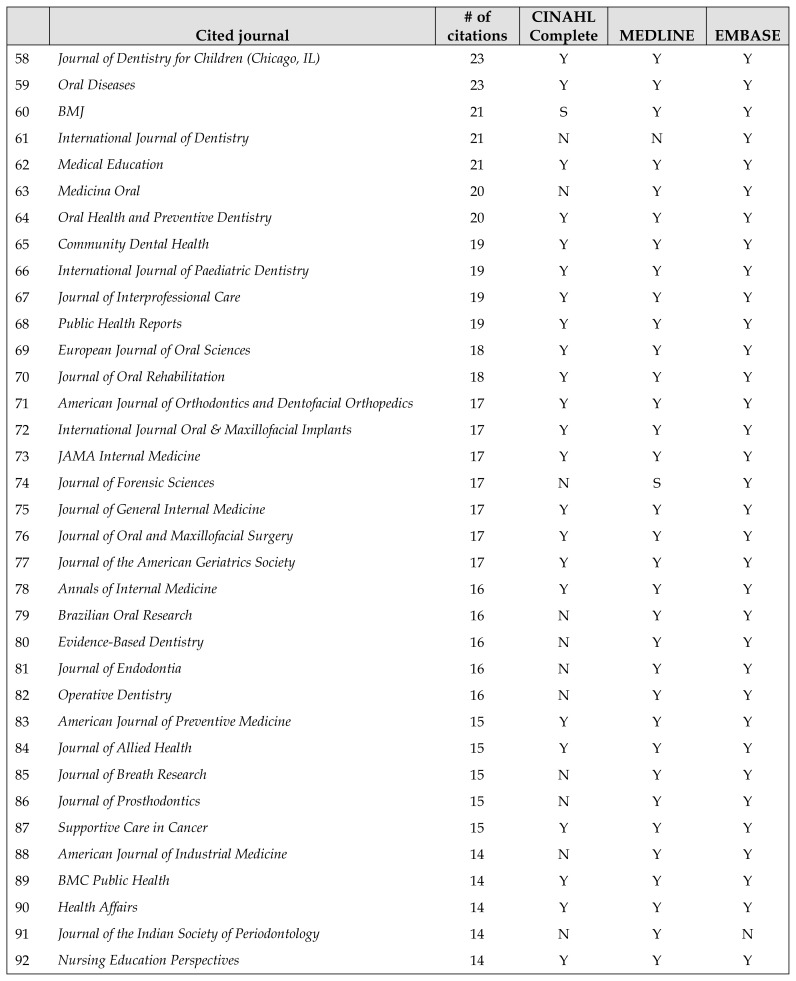
Distribution and indexing coverage in of cited journals in Zone 1 and Zone 2

	Cited journal	# of citations	CINAHL Complete	MEDLINE	EMBASE
	**Zone 1**				
1	*Journal of Periodontology*	473	Y	Y	Y
2	*Journal of Clinical Periodontology*	409	Y	Y	Y
3	*Journal of the American Dental Association*	390	Y	Y	Y
4	*Journal of Dental Hygiene*	370	Y	Y	Y
5	*Journal of Dental Education*	348	Y	Y	Y
6	*Dimensions of Dental Hygiene*	228	Y	N	N
7	*Journal of Dental Research*	198	Y	Y	Y
8	*International Journal of Dental Hygiene*	146	Y	Y	Y
9	*International Journal of Clinical Pediatric Dentistry*	111	Y	Y	Y
10	*Periodontology 2000*	103	Y	Y	Y
11	*Cochrane Database of Systematic Reviews*	91	N	Y	Y
	**Total indexing coverage for Zone 1**		**90.9%**	**90.9%**	**90.9%**
	**Zone 2**				
12	*Community Dentistry and Oral Epidemiology*	90	Y	Y	Y
13	*Journal of the California Dental Association*	84	Y	N	N
14	*Journal of Public Health Dentistry*	74	Y	Y	Y
15	*Oral Surgery, Oral Medicine, and Oral Pathology*	74	Y	Y	Y
16	*British Dental Journal*	69	N	Y	Y
17	*Caries Research*	61	Y	Y	Y
18	*Dental Clinics of North America*	58	Y	Y	Y
19	*International Dental Journal*	58	Y	Y	Y
20	*Clinical Oral Implants Research*	56	N	Y	Y
21	*American Journal of Public Health*	55	Y	Y	Y
22	*Diabetes Care*	51	Y	Y	Y
23	*Journal of Dentistry*	51	Y	Y	Y
24	*New England Journal of Medicine*	51	Y	Y	Y
25	*Journal of Clinical Dentistry*	43	Y	Y	Y
26	*BMC Oral Health*	42	Y	Y	Y
27	*Clinical Oral Investigations*	42	N	Y	Y
28	*Journal of Evidence-Based Dental Practice*	42	Y	Y	Y
29	*Australian Dental Journal*	40	Y	Y	Y
30	*Journal of the Canadian Dental Association*	39	Y	Y	Y
31	*Canadian Journal of Dental Hygiene*	38	Y	N	N
32	*Lancet*	38	Y	Y	Y
33	*Special Care in Dentistry*	38	Y	Y	Y
34	*Morbidity and Mortality Weekly Report*	37	Y	Y	Y
35	*Access*	36	Y	N	N
36	*PLoS One*	36	N	Y	Y
37	*Quintessence International*	36	Y	Y	Y
38	*American Journal of Dentistry*	35	N	Y	Y
39	*General Dentistry*	35	Y	Y	Y
40	*Journal of Periodontal Research*	35	Y	Y	Y
41	*Pediatrics*	35	Y	Y	Y
42	*Annals of Periodontology*	34	Ceased	Ceased	Ceased
43	*JAMA*	34	Y	Y	Y
44	*Acta Odontologica Scandinavica*	32	Y	Y	Y
45	*Anesthesia Progress*	32	Y	Y	Y
46	*Compendium of Continuing Education in Dentistry*	32	Y	Y	N
47	*Morbidity and Mortality Weekly Report: Recommendations and Reports*	30	Y	Y	Y
48	*Gerodontology*	29	Y	Y	Y
49	*International Journal of Periodontics and Restorative Dentistry*	29	Y	Y	Y
50	*Journal of Prosthetic Dentistry*	29	Y	Y	Y
51	*Work*	26	Y	Y	Y
52	*European Journal Dental Education*	25	N	Y	Y
53	*Academic Medicine*	24	N	Y	Y
54	*Archives of Oral Biology*	24	N	Y	Y
55	*Circulation*	24	Y	Y	Y
56	*Dental Traumatology*	23	Y	Y	Y
57	*Journal of Advanced Nursing*	23	Y	Y	Y
58	*Journal of Dentistry for Children (Chicago, IL)*	23	Y	Y	Y
59	*Oral Diseases*	23	Y	Y	Y
60	*BMJ*	21	S	Y	Y
61	*International Journal of Dentistry*	21	N	N	Y
62	*Medical Education*	21	Y	Y	Y
63	*Medicina Oral*	20	N	Y	Y
64	*Oral Health and Preventive Dentistry*	20	Y	Y	Y
65	*Community Dental Health*	19	Y	Y	Y
66	*International Journal of Paediatric Dentistry*	19	Y	Y	Y
67	*Journal of Interprofessional Care*	19	Y	Y	Y
68	*Public Health Reports*	19	Y	Y	Y
69	*European Journal of Oral Sciences*	18	Y	Y	Y
70	*Journal of Oral Rehabilitation*	18	Y	Y	Y
71	*American Journal of Orthodontics and Dentofacial Orthopedics*	17	Y	Y	Y
72	*International Journal Oral & Maxillofacial Implants*	17	Y	Y	Y
73	*JAMA Internal Medicine*	17	Y	Y	Y
74	*Journal of Forensic Sciences*	17	N	S	Y
75	*Journal of General Internal Medicine*	17	Y	Y	Y
76	*Journal of Oral and Maxillofacial Surgery*	17	Y	Y	Y
77	*Journal of the American Geriatrics Society*	17	Y	Y	Y
78	*Annals of Internal Medicine*	16	Y	Y	Y
79	*Brazilian Oral Research*	16	N	Y	Y
80	*Evidence-Based Dentistry*	16	N	Y	Y
81	*Journal of Endodontia*	16	N	Y	Y
82	*Operative Dentistry*	16	N	Y	Y
83	*American Journal of Preventive Medicine*	15	Y	Y	Y
84	*Journal of Allied Health*	15	Y	Y	Y
85	*Journal of Breath Research*	15	N	Y	Y
86	*Journal of Prosthodontics*	15	N	Y	Y
87	*Supportive Care in Cancer*	15	Y	Y	Y
88	*American Journal of Industrial Medicine*	14	N	Y	Y
89	*BMC Public Health*	14	Y	Y	Y
90	*Health Affairs*	14	Y	Y	Y
91	*Journal of the Indian Society of Periodontology*	14	N	Y	N
92	*Nursing Education Perspectives*	14	Y	Y	Y
93	*American Journal of Pharmaceutical Education*	13	Y	Y	Y
94	*DentoMaxilloFacial Radiology*	13	N	N	Y
95	*International Journal of Prosthodontontics*	13	Y	Y	Y
96	*Journal of Clinical and Diagnostic Research*	13	N	N	Y
97	*Journal of Contemporary Dental Practice*	13	Y	Y	Y
98	*Journal of Nursing Education*	13	Y	Y	Y
99	*Journal of the International Academy of Periodontology*	13	Y	N	N
100	*New York State Dental Journal*	13	Y	N	N
101	*Nurse Education Today*	13	Y	Y	Y
102	*Patient Education and Counseling*	13	Y	Y	Y
103	*Clinical Infectious Diseases*	12	Y	Y	Y
104	*Dental Materials*	12	Y	Y	Y
105	*Journal of Clinical Pediatric Dentistry*	12	Y	Y	Y
106	*Journal of Occupational and Environmental Medicine*	12	Y	Y	Y
107	*Journal of Oral Pathology & Medicine*	12	Y	Y	Y
108	*Nicotine & Tobacco Research*	12	Y	Y	Y
109	*Obstetrics and Gynecology*	12	Y	Y	Y
110	*Indian Journal Dental Research*	11	Y	Y	Y
111	*Journal of Esthetic and Restorative Dentistry*	11	Y	Y	Y
112	*Journal of the Academy of Nutrition and Dietetics*	11	Y	Y	Y
113	*Academic Pediatrics*	10	Y	Y	Y
114	*American Journal of Respiratory and Critical Care Medicine*	10	Y	Y	Y
115	*Journal of Applied Oral Science*	10	N	Y	Y
116	*Morbidity and Mortality Weekly Report: Surveillance Summaries*	10	Y	Y	Y
117	*Nurse Educator*	10	Y	Y	Y
118	*Open Dentistry Journal*	10	N	N	N
119	*Preventing Chronic Disease*	10	Y	Y	Y
	**Total indexing coverage for Zones 1 and 2**		**78.2%**	**89.9%**	**91.6%**

## DISCUSSION

The most striking finding was the sheer volume of work that has been produced since the 1999 dental hygiene mapping study was performed. Previously, a small core of only 5 titles constituted Zone 1, which has now grown to 11 titles. In 1999, Zone 2 comprised 42 journals, which number has grown to 108. The total number of journals referenced has grown more than 4-fold from 389 to 1,675.

Citation patterns may have been skewed by the fact that over half the citations came from *Dimensions of Dental Hygiene*, a journal that is highly practice oriented. The increased number of journal titles cited may be due to the use of web search engines, such as Google Scholar, for scholarly research. However, the 2 time periods are not strictly comparable due to the greater number of source articles (426 versus 149) and the larger number of citations (10,901 versus 2,632) in the present study.

Bradford’s Law of Scattering, a unifying feature of NAHRS mapping studies, is described as “most valuable to librarians who are faced with the cost-benefit considerations of additional journal coverage” [11]. Based on Bradford’s classic, *Documentation* [12], this law postulates that journals in a discipline can be divided into three zones, each containing one-third of the references cited by writers in that discipline. Its utility for librarians lies in the assumption that Zone 1 contains a small number of core journals, which could be considered a library’s “must have” titles. Zone 2 is a larger number of “nice to have” journals, and Zone 3 contains an even larger number of titles to which one need not subscribe but which one may be asked to obtain. Because 78.4% of citations came from journals and one-third of these came from 11 core journals, the notion of “core” journals still seems viable.

Traditional refereed journals have retained their preeminent place in dental hygiene research. In fact, the percentage of journal article citations increased from 69.5% of total cited references in the 1999 study [2] to 78.4% in the present study. Moreover, the same 5 journals occupied the top spots in both the 1999 and the present study, albeit in different order: *Journal of Dental Hygiene*, *Journal of the American Dental Association*, *Journal of Periodontology*, *Journal of Dental Education*, and *Journal of Clinical Periodontology*. All Zone 1 journals were refereed, and all but *Dimensions of Dental Hygiene* are subscription-based and might be less accessible to dental hygienists outside an academic setting.

Two journal titles that began in 2003 now appear in Zone 1: *Dimensions of Dental Hygiene*, a refereed, practice-oriented title freely available on the web, and the *International Journal of Dental Hygiene*, a more research-oriented journal. Another interesting addition to Zone 1 is the *Cochrane Database of Systematic Reviews*, which began in the 1990s and was cited ninety-one times. In all, Zone 1 contained seven dental titles, three dental hygiene titles, and the *Cochrane Database of Systematic Reviews*. The Zone 1 journal list provides a good answer to the frequently asked question, “What are the ‘top’ journals in my field to which I should submit my manuscript?”

A key development in dental hygiene research from 1999 to 2018 was the growth of the World Wide Web. In 1995, CompuServe and America Online began marketing dial-up access to end users [13], but most research literature was still used in print format. The 1999 mapping study [2] did not even mention the web or evidence-based practice. The present study, like other recent mapping studies, considers a fifth format type, “Internet sources,” which accounted for 8.1% of citations. A more accurate designation for this format type might be “Internet sources, not otherwise specified,” as most journal articles and government publications, and many books are now consulted online.

One might expect the cited references in the present study to be more current than in the 1999 study [2], as online publishing allows quicker access, but this was not the case for journal articles. Interestingly, in the present study, only 64.8% of articles cited were published in the previous 10 years, compared with 71.1% in 1999 [2]. Perhaps researchers are partially switching to Internet sites or government publications for current information. Most Internet sources (72.1%) and government documents (71.8%) were 5 years old or less when they were cited. Conversely, historical sources were conspicuously absent from this sample. In both the 1999 study [2] and the present study, there was little use of materials that were older than 25 years. Only 3.8% of citations in 1999 [2] and 6.2% in the present study were more than 25 years old.

Book citations, including both print and electronic books, shrank from 18.1% of total citations in 1999 [2] to 5.1% in the present study. This might be because researchers now browse the web for information that used to be found in books. Items classified as “miscellaneous,” which decreased sharply from 7.4% in 1999 [2] to 1.6% in the present study, might also have migrated to the web. Government publications increased slightly, from 5.0% in 1999 [2] to 6.5% in the present study.

Improved database coverage and web search engines appear to have occurred in parallel with an increase in dental hygiene research between 1999 and the present. In 1999 [2], only MEDLINE was deemed to index the dental hygiene field adequately. EMBASE covered less than one-fourth of titles in 1999 [2] but covered the majority (91.6%) of titles in the present study. Also CINAHL indexed only 23.4% of titles in 1999 [2], but CINAHL Complete, the “premier” version of CINAHL, now covers 78.2% of titles. Other versions of CINAHL that are still used in many locations cover fewer titles. The results of this study suggest that CINAHL should be combined with another database, such as MEDLINE, to search the dental hygiene literature, because CINAHL has several unique titles, as do MEDLINE and EMBASE. However, the database indexing coverage results should be used with caution for several reasons. For one, the present coverage figures are based on vendor self-report rather than on direct checking as was done in 1999 [2]. Also, unlike the 1999 study [2], the present study did not attempt to ascertain the level of indexing.

Marked changes in database access between 1999 and the present also deserve mention. In the 1990s, researchers commonly searched individual databases on locally mounted discs, through librarian-mediated searches, or via print indexes. Today, most searches are done on the web using open access or subscription databases, or web browsers such as Google and Google Scholar. Also, multiple databases are often searched together via library federated search engines or vendor-supplied packages. In addition to database searching, dental hygienists commonly access several key titles directly. For example, *Dimensions of Dental Hygiene* is freely available online and is distributed free of charge to students. Additionally, the *Journal of Dental Hygiene* and *Access* are widely available to members of the American Dental Hygiene Association.

Compared with the 1999 mapping study [2], the list of journals referenced by dental hygiene authors has grown more than 4-fold. Compared with 1999, book citations have decreased, government publication citations have increased slightly, and Internet source citations have appeared. The self-reported coverage of Zone 1 and Zone 2 journals for CINAHL Complete, MEDLINE, and EMBASE was 78.2%, 89.9%, and 91.6%, respectively. Researchers also cite both journal articles and miscellaneous resources (“grey literature”) such as professional association statements, dissertations, and conference presentations. These findings suggest that dental hygiene research is growing, and indexing coverage for this field has improved dramatically in the past two decades. As research in dental hygiene grows, librarians can use the results of this study to advise dental hygiene researchers as to which databases to use and to which journals to submit their work.
